# Evaluation of Biomarkers and Immune Microenvironment of Osteoarthritis: Evidence From Omics Data and Machine Learning

**DOI:** 10.3389/fgene.2022.905027

**Published:** 2022-05-16

**Authors:** Zhixin Liu, Heng Liu, Deqiang Li, Liang Ma, Tongxin Lu, Hao Sun, Yuankai Zhang, Hui Yang

**Affiliations:** ^1^ Department of Orthopedics, Qilu Hospital of Shandong University, Jinan, China; ^2^ NHC Key Laboratory of Otorhinolaryngology, Qilu Hospital of Shandong University, Jinan, China; ^3^ Department of Otorhinolaryngology, Qilu Hospital of Shandong University, Jinan, China; ^4^ Department of Radiology, Qilu Hospital of Shandong University, Jinan, China

**Keywords:** osteoarthritis, immune microenvironment, biomarkers, machine learning, OMICS data

## Abstract

**Objectives:** This study aimed to identify novel biomarkers for osteoarthritis (OA) and explore potential pathological immune cell infiltration.

**Methods:** We identified differentially expressed genes (DEGs) between OA and normal synovial tissues using the *limma* package in R, and performed enrichment analyses to understand the functions and enriched pathways of DEGs. Weighted gene co-expression network analysis (WGCNA) and distinct machine-learning algorithms were then used to identify hub modules and candidate biomarkers. We assessed the diagnostic value of the candidate biomarkers using receiver operating characteristic (ROC) analysis. We then used the CIBERSORT algorithm to analyze immune cell infiltration patterns, and the Wilcoxon test to screen out hub immune cells that might affect OA occurrence. Finally, the expression levels of hub biomarkers were confirmed by quantitative reverse transcription-polymerase chain reaction (qRT-PCR).

**Results:** We identified 102 up-regulated genes and 110 down-regulated genes. The functional enrichment analysis results showed that DEGs are enriched mainly in immune response pathways. Combining the results of the algorithms and ROC analysis, we identified GUCA1A and NELL1 as potential diagnostic biomarkers for OA, and validated their diagnosibility using an external dataset. Construction of a TF-mRNA-miRNA network enabled prediction of potential candidate compounds targeting hub biomarkers. Immune cell infiltration analyses revealed the expression of hub biomarkers to be correlated with CD8 T cells, memory B cells, M0/M2 macrophages, resting mast cells and resting dendritic cells. qRT-PCR results showed both GUCA1A and NELL1 were significantly increased in OA samples (*p* < 0.01). All validations are consistent with the microarray hybridization, indicating that GUCA1A and NELL1 may be involved in the pathogenesis of OA.

**Conclusion:** The findings suggest that GUCA1A and NELL1, closely related to OA occurrence and progression, represent new OA candidate markers, and that immune cell infiltration plays a significant role in the progression of OA.

## Introduction

Characterized by cartilage degeneration, sclerosis of subchondral bone and osteophyte formation, osteoarthritis (OA) is the most common degenerative joint disease (Mathiessen and Conaghan, 2017). Patients with OA experience chronic pain, swelling, malformation and joint stiffness, which may lead to progressive disability and deterioration of patients’ quality of life (Parkinson et al., 2017). It is estimated that approximately 9.6% of men and 18% of women worldwide aged 60 years or over suffer from OA, and by 2030 nearly 67 million people living in the United States will have been diagnosed with the disease (Hootman and Helmick, 2006; Li et al., 2017). Unfortunately, current OA therapies cannot prevent or reverse the progress of the disease, and are limited to inhibiting pain and alleviating inflammation (Seed et al., 2011). Advanced patients undergo joint replacement surgery.

Considerable attention has been given to identifying promising biomarkers for disease diagnosis and therapy through transcriptomic and microarray analyses (Demircioğlu et al., 2019; Carr et al., 2020). A noteworthy study has found that the m6A demethylase FTO, which plays a tumor-suppressing role, may be a prospective risk biomarker for thyroid cancer (Tian et al., 2020). Based on the Gene Expression Omnibus (GEO) database, GZMA, PRC1 and TTK were enriched in the innate immune cell-mediated immune response and immune-related biological processes, validating them as potential targets for rheumatoid arthritis (RA) therapy (Cheng et al., 2021). It has also been reported that IFI27 may play a vital role in the occurrence of systemic lupus erythematosus (SLE), and may be a possible target for SLE diagnosis (Zhao et al., 2021). Therefore, it is vital to explore the molecular mechanisms underlying the development and progression of OA, and to identify new and effective biomarkers for its diagnosis and treatment.

In this study, we first acquired differentially expressed genes (DEGs) in OA and normal synovial tissue by mining four GEO datasets (GSE55235, GSE55457, GSE12021 and GSE82107). Next, we conducted a series of enrichment analyses of functions and pathways for these DEGs. To evaluate the key module and to screen out hub biomarkers highly correlated with OA, we performed weighted gene co-expression network analysis (WGCNA) and applied three machine-learning algorithms: least absolute shrinkage and selection operator (LASSO), support vector machine-recursive feature elimination (SVM-RFE) and logistic regression. We validated the selected hub genes using GEO datasets (GSE89408), and verified their diagnostic value with receiver operating characteristic (ROC) curves. A TF-mRNA-miRNA network was then constructed, and potential candidate compounds targeting the biomarkers were predicted. We used the CIBERSORT algorithm and the Wilcoxon test to analyze the difference in immune infiltration between OA and normal tissues and the relationship between biomarkers and infiltrating immune cells, and to identify hub immune cells that might affect OA. Finally, the expression levels of hub biomarkers were confirmed by qRT-PCR. This study strengthens understanding of the mechanisms of development and pathogenesis in OA at the transcriptome level, and provides new insights into potential biomarkers for diagnosis and treatment of OA.

## Materials and Methods

### Data Collection

Gene expression profiles of OA and normal synovial tissue were downloaded from the GEO database (https://www.ncbi.nlm.nih.gov/geo/) (Barrett et al., 2013). To be eligible for selection, the profiles must have been produced with *Homo sapiens* expression profile analysis using array, and be of OA or normal synovial tissue from joint synovial biopsies, the datasets must contain more than five samples and complete sample information, and each subject had to have one biopsy sample analyzed without duplication. Three GPL96 datasets (GSE55235, GSE55457 and GSE12021), and GSE82107 based on the GPL570 platform, were selected as test sets, including 40 OA samples and 36 normal samples. We downloaded the original GSE89408 count data, a dataset based on the GPL11154 platform, as a validation set (22 OA and 28 normal synovial tissue samples). Patients’ clinical features are detailed in Supplementary Material S1.

### Data Processing and Identification of DEGs

The datasets were combined, and the *sva* package (Leek et al., 2012) was used to normalize the original data (Supplementary Figure S1). The DEGs were screened in the batch calibrated test set using the *limma* package (Ritchie et al., 2015). We selected | log2 fold change FC | > 1 and adj. p. value < 0.05 as truncation criteria.

### Functional Enrichment Analyses

The *GOplot* program package (Walter et al., 2015) was used to visualize the gene ontology (GO), Kyoto Encyclopedia of Genes and Genomes (KEGG) pathway and disease ontology (DO) analysis. Terms and pathways with *p* < 0.05 were considered statistically significant. We used the *clusterprofiler* R package (Yu et al., 2012) to conduct GSEA on the DEGs using sequencing data, and the *GSVA* R program (Hänzelmann et al., 2013) to identify pathways most closely associated with DEGs, with *p* values <0.05 being considered statistically significant. The h. all.v7.4. symbols gene set was downloaded from MSigDB (Liberzon et al., 2015), and GSEA analysis was performed on the gene set and gene expression matrix to explore possible regulatory pathways involved.

### WGCNA Network Analysis and Key Module Identification

A co-expression network targeting DEGs was constructed using the *WGCNA* package (Langfelder and Horvath, 2008). In WGCNA analysis, all DEGs with an adjusted *p* value <0.05 and | log2 fold change FC | > 1 in the OA and normal samples were taken as inputs for topology calculation, with soft threshold values ranging from 1 to 20. The *β* value is determined from the lowest value near scale-free network, and the optimal soft threshold was determined to be 8. Following the optimal soft threshold, the relation matrix was converted into an adjacency matrix and then into a topological overlap matrix (TOM). We carried out average link hierarchy clustering, and classified relevant modules according to the TOM, with the number of genes in each module being no less than 50. Similar modules were then merged. The Pearson method was used to calculate correlation between the combined module and OA, and hub modules and potential hub genes relating to clinical traits were identified.

### Machine Learning-Based Hub Biomarker Screening

Machine-learning classification algorithms are increasingly being used to predict feature genes associated with diseases. LASSO (Engebretsen and Bohlin, 2019) is a regression analysis method for both gene selection and gene classification. In order to avoid collinearity generated by high-dimensional data, redundant genes were eliminated using LASSO’s 10-fold cross validation (*GLMNET* package) on the genes screened by WGCNA. SVM-RFE (Yoon and Kim, 2009; Lin et al., 2012) is a machine-learning method based on the support vector machine (SVM), which finds the optimal variable by subtracting the feature vector generated by SVM. The SVM-RFE method was then used on the genes processed by LASSO for further screening to produce the optimal number of genes, while minimizing classification errors and overfitting. We then used univariate logistic regression analysis to screen the genes, with *p* < 0.001 as the threshold. Finally, the DEGs, SVM-RFE-screened genes and logistic regression-screened genes were overlapped to identify hub biomarkers.

### Validation of Hub Biomarkers

Expression analysis of the hub biomarkers was performed on the test set. The ROC curves were then plotted using the *pROC* R package (Robin et al., 2011), and the area under the curve (AUC) was calculated separately to evaluate the predictive utility of identified hub genes. Values of AUC >0.7 and *p* < 0.05 indicated that the genes were highly predictive for OA diagnosis. The validation set GSE89408 based on the GPL11154 platform was used to verify the analysis results.

### Construction of Regulatory Network

The mirDIP database (Tokar et al., 2018) was used to predict the potential miRNA of targeted hub genes and identify the miR regulatory network. TF-hub gene interactions with *p* values <0.05 were selected from the TRRUST database (Han et al., 2018) to establish upstream regulatory networks. In addition, compounds with potential relationships to hub genes were searched in the Comparative Toxicogenomics database (Davis et al., 2019). Finally, the hub genes regulatory network was visualized based on the Networkanalyst database (Zhou et al., 2019).

### Analysis of Immune Cell Infiltration, Correlations Between Hub Genes and Immune Cell Infiltration

The CIBERSORT algorithm (Chen et al., 2018) was used to calculate the proportions of different immune cell types, according to the expression levels of immune cell-related genes. The output results for 22 infiltrated immune cell types were integrated to generate an immune cell component matrix for analysis. Relationships between hub biomarkers’ expression levels and immune cell infiltration were examined using Pearson’s rank correlation analyses, conducted and visualized with the *ggpubr* R package.

### Identification of Hub Immune Cells

The Wilcoxon test was used to investigate differences in immune cell content between different tissues. The random forest program package was used to construct a random forest tree of the 22 kinds of immune cells to identify the point with the minimum error, and the immune cells were ranked by importance. Genes with importance scores greater than two were selected for screening. The identified immune cells were overlapped to screen out hub immune cells that might affect the occurrence of OA.

### qRT-PCR Validation of Hub Biomarkers

In order to confirm the results of bioinformatics analysis, we collected synovial tissues from 10 OA patients and 10 patients without OA for qRT-PCR verification. The study protocol was approved by the ethics committee of Qilu Hospital of Shandong University, and all patients signed informed consent. Total RNA was extracted from synovial tissue using TRIzol^®^ Reagent (15596026, Thermo Fisher Scientific, Inc.). An qRT-PCR kit (K1005S, Promega Biotech Co.) was used to synthesize the first strand of cDNA from equal amounts of total RNA samples, and real-time fluorescence PCR was performed with SYBR Green Realtime PCR Master Mix (QPK-201, TOYOBO Co., Ltd., Kita-ku, Osaka, Japan) according to the manufacturer’s protocol. We selected ß-actin as the inner control and employed the 2^−ΔΔCt^ method to quantify the relative mRNA level. The sequences of NELL1 were as follows: TCA​CAG​GAA​GCC​ACT​GCG​AGA​A (sense) and CCA​TCG​TCA​TGG​AAA​CCG​CTT​C (antisense). The sequences of GUCA1A were as follows: GCA​GAG​GAG​TTC​ACC​GAT​ACA​G (sense) and GTC​AGT​GTG​TCC​AGG​AGC​ATC​T (antisense). The sequences of *ß*-actin were as follows: CAC​CAT​TGG​CAA​TGA​GCG​GTT​C (sense) and AGG​TCT​TTG​CGG​ATG​TCC​ACG​T (antisense). One-way analysis of variance was used for the statistical analysis, and *p* < 0.05 indicated a significant difference.

## Results

### Differentially Expressed Genes Between OA and Normal Synovial Tissues

**FIGURE 1:**
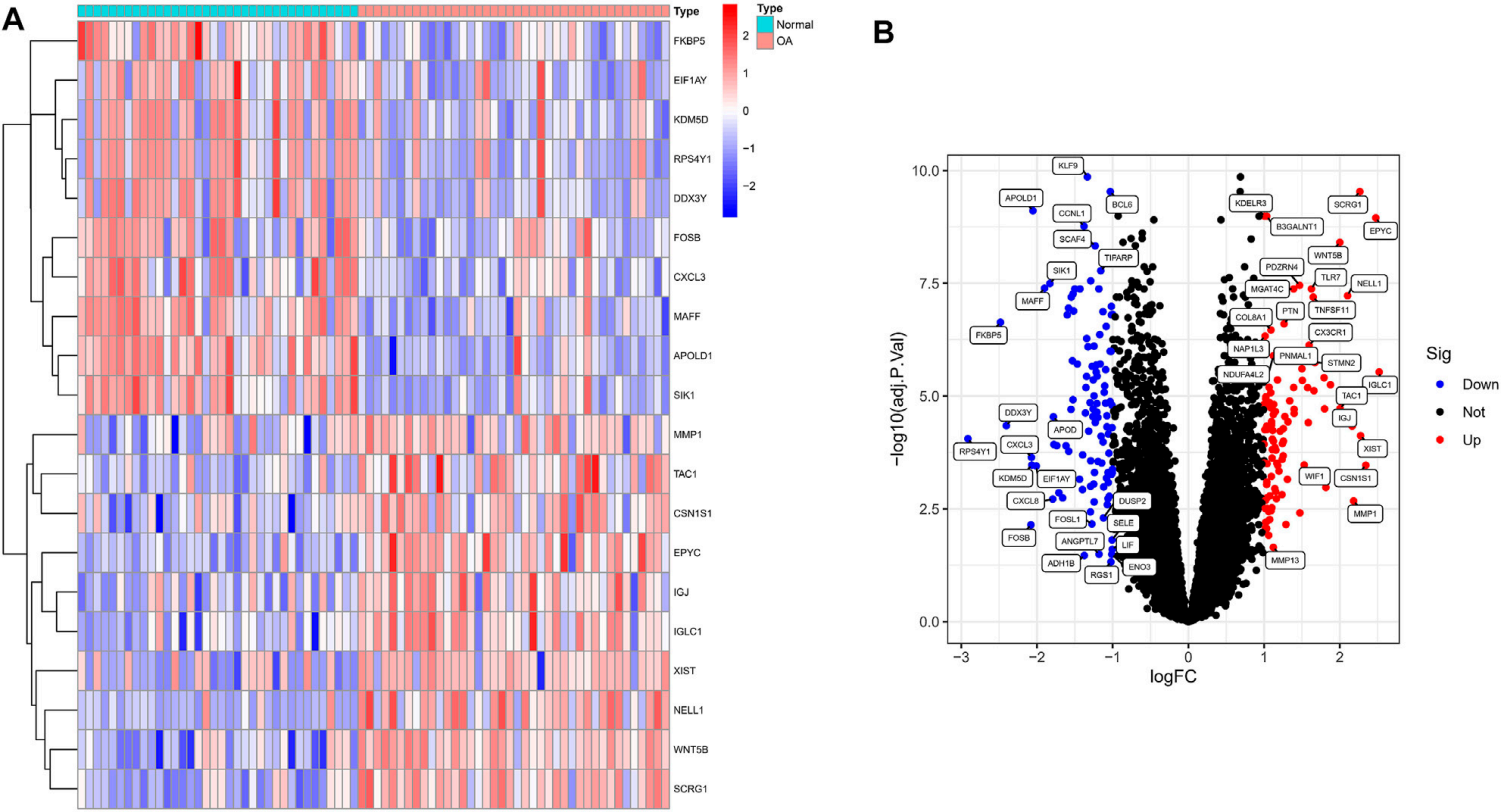
Identification of significant differentially expressed genes (DEGs) in OA. **(A)** Heatmap of DEGs between OA and normal samples. **(B)** Volcano plot of DEGs between OA and normal samples. Red rectangles/plots represent up-regulated genes and blue rectangles/plots represent down-regulated genes.

We analyzed the DEGs of 40 OA and 36 normal synovium samples in the test set (GSE55235, GSE55457, GSE12021 and GSE82107 datasets), and identified a total of 212 DEGs in the OA samples compared with the normal group (Supplementary Material S2). Figure 1A shows a heat map of the top 20 differential genes by log fold change, and the volcano map in Figure 1B illustrates that 102 genes were significantly up-regulated and 110 significantly down-regulated.

### Enrichment Analysis of DEGs

**FIGURE 2:**
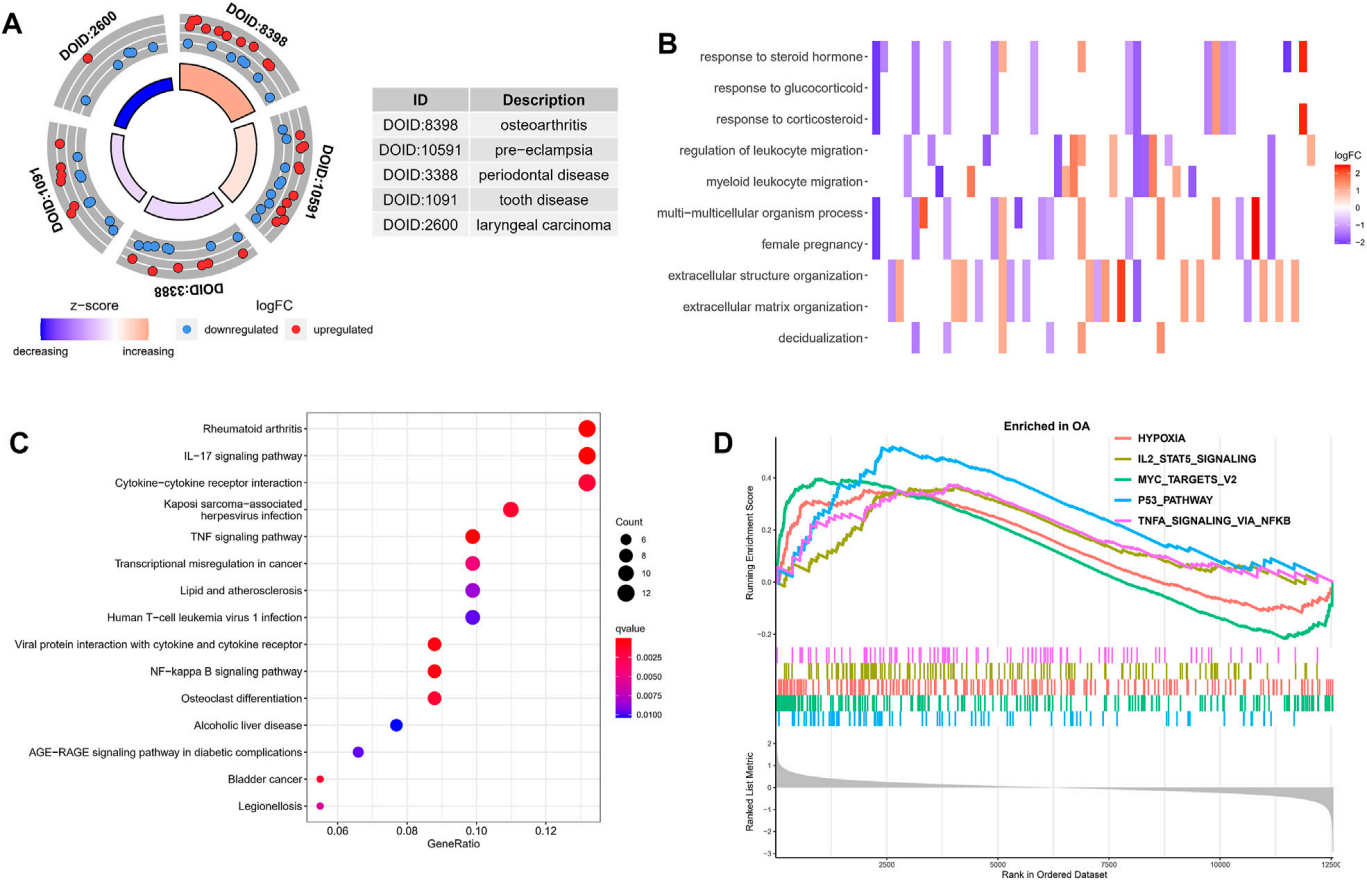
Functional enrichment analysis of DEGs. **(A)** DO analysis results for disease types that may share a common pathogenesis with OA. **(B)** The top 10 enriched biological processes of DEGs identified using GO analysis. **(C)** KEGG enrichment analysis based on the DEGs. The gradual bubble color represents the adjusted *p* value, and the bubble size represents the gene count. **(D)** GSEA analysis of DEGs in the OA and normal groups.

Next, in order to explore the potential biological mechanism of OA progression, we observed the enrichment pathway of DEGs from multiple perspectives. DO analysis revealed disease types that may share a common pathogenesis with OA, such as pre-eclampsia, periodontal disease and dental disease (Figure 2A). GO enrichment analysis of the DEGs showed that the immune response in OA samples was stronger than in the normal sample, including regulation of leukocyte migration and myeloid leukocyte migration. The top 10 biological processes were selected, with *Q* values <0.05, as shown in Figure 2B. KEGG pathway enrichment analysis showed related genes involved in, for example, the IL-17 signaling pathway, cytokine-cytokine receptor interaction and the TNF signaling pathway (Figure 2C). These results indicated that immune-related factors may affect the progression of OA, GSEA analysis was performed on the gene set and expression matrix. The results demonstrated that hypoxia, IL-2-STAT5 signaling and other pathways play an important role (Figure 2D). These strong evidence chains suggest that OA may be regulated by immune pathways.

### Further Screening With WGCNA Analysis

**FIGURE 3:**
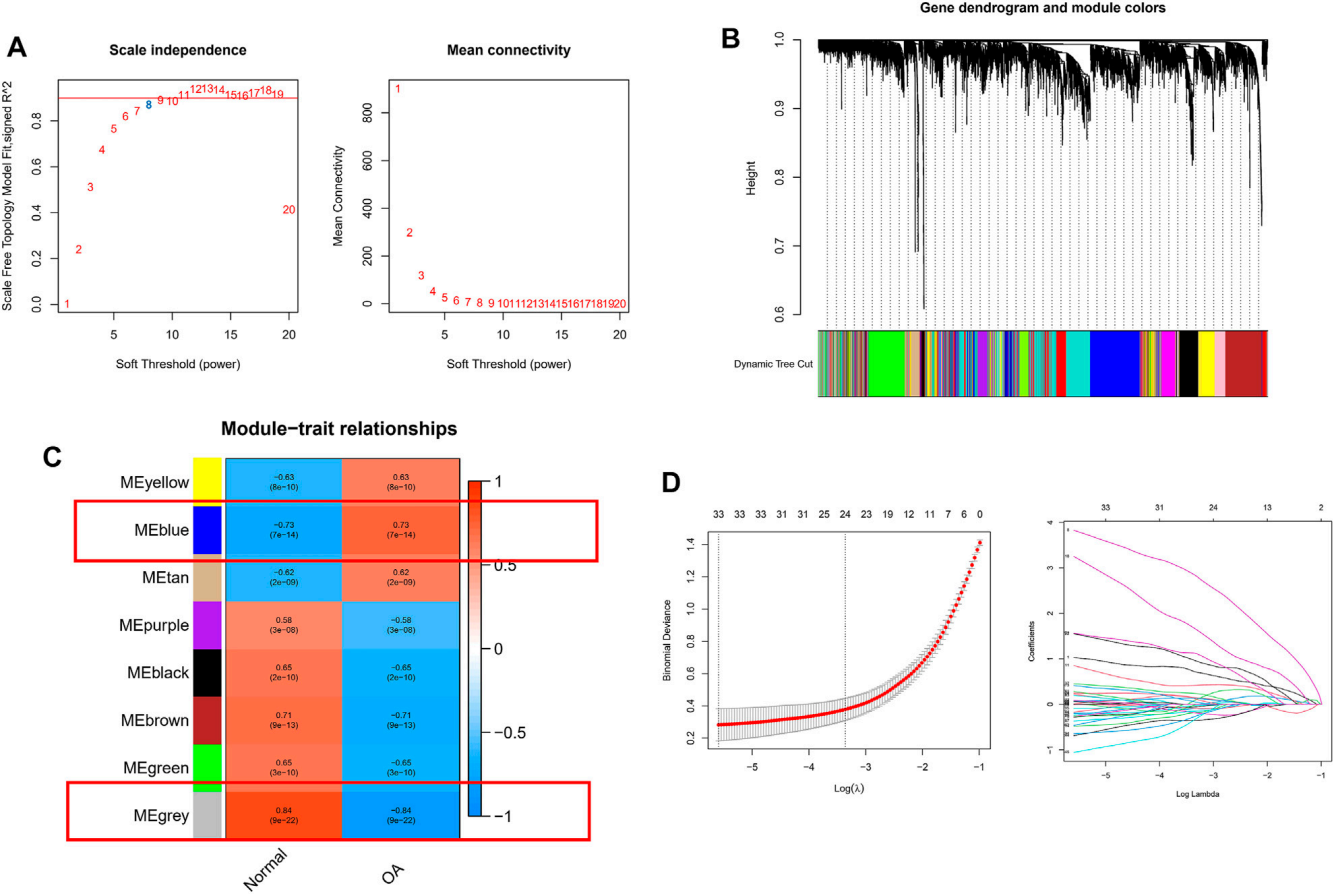
WGCNA-based identification of co-expression modules from merged datasets. **(A)** Soft thresholds (*β*) and scale-free topology fitting indices (*R* ^2^). To maximize model fit, a *β* value of eight was chosen. **(B)** Dendrograms generated via average linkage hierarchical clustering of identified modules. **(C)** Associations between modules’ clinical status in normal and OA patient samples, with individual rows corresponding to module eigengenes and columns corresponding to clinical characteristics. Correlations and *p* values are shown in the first and second lines of each cell, respectively. **(D)** Coefficient profile plot generated against the log(lambda) sequence, using the LASSO logistic regression algorithm to screen diagnostic markers. Different colors represent different genes.

To further correlate clinical information with key genes, we performed WGCNA analysis. The clustering of each sample was good, with no outlier samples. A soft threshold from 1 to 20 was used for topology calculation, and the optimal soft threshold was determined to be 8 (Figure 3A). Using the soft threshold, the relational matrix was transformed into an adjacency matrix, and then into a topological overlap matrix (TOM) to determine average link hierarchical clustering. Related modules were classified according to the TOM, with the number of genes in each module being no less than 50 (Figure 3B). Similar gene modules were merged, resulting in eight modules (Figure 3C). Correlation between genes and clinical traits in the module was calculated, revealing that the blue module containing 1,776 genes exhibited the highest positive correlation with OA occurrence (r = 0.73), and the grey module containing 128 genes had the highest negative correlation with OA occurrence (R = −0.84). A total of 1,904 potential core genes were identified.

### Exploration of Hub Biomarkers

**FIGURE 4:**
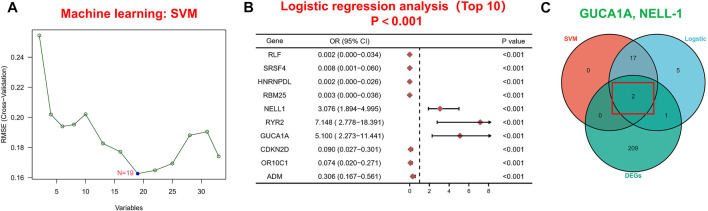
Machine learning-based hub biomarker screening. **(A)** Diagnostic marker screening using the SVM-RFE algorithm. **(B)** The 10 most significant genes identified by univariate logistic analysis (*p* < 0.001). **(C)** Overlapping genes predicted by the DEGs, logistic regression and SVM-RFE algorithms.

Next, we applied a series of machine-learning algorithms—LASSO, SVM-RFE and logistic regression—to screen the most significant genes associated with OA. A total of 1,904 potential hub genes screened by WGCNA in OA patients were selected between the two groups to fit the LASSO regression model. The next step was to use LASSO’s 10-fold cross-validation to remove any further redundant genes, as a result of which 33 potential genes with non-zero coefficients identified in OA and normal cohorts were screened out (Figure 3D). We used the SVM-RFE algorithm for in-depth screening of these 33 genes. The results showed that the RMSE value was lowest when 19 genes were selected as variables (Figure 4A). Taking occurrence of OA as the dependent variable, univariate logistic analysis was then carried out. The forest map produced 25 genes with *p* values less than 0.001 (Figure 4B). Finally, we overlapped the genes of the two previous identification algorithms with 212 DEGs, and identified GUCA1A and NELL1 as our hub biomarkers (Figure 4C).

### Validation of Hub Biomarkers

**FIGURE 5:**
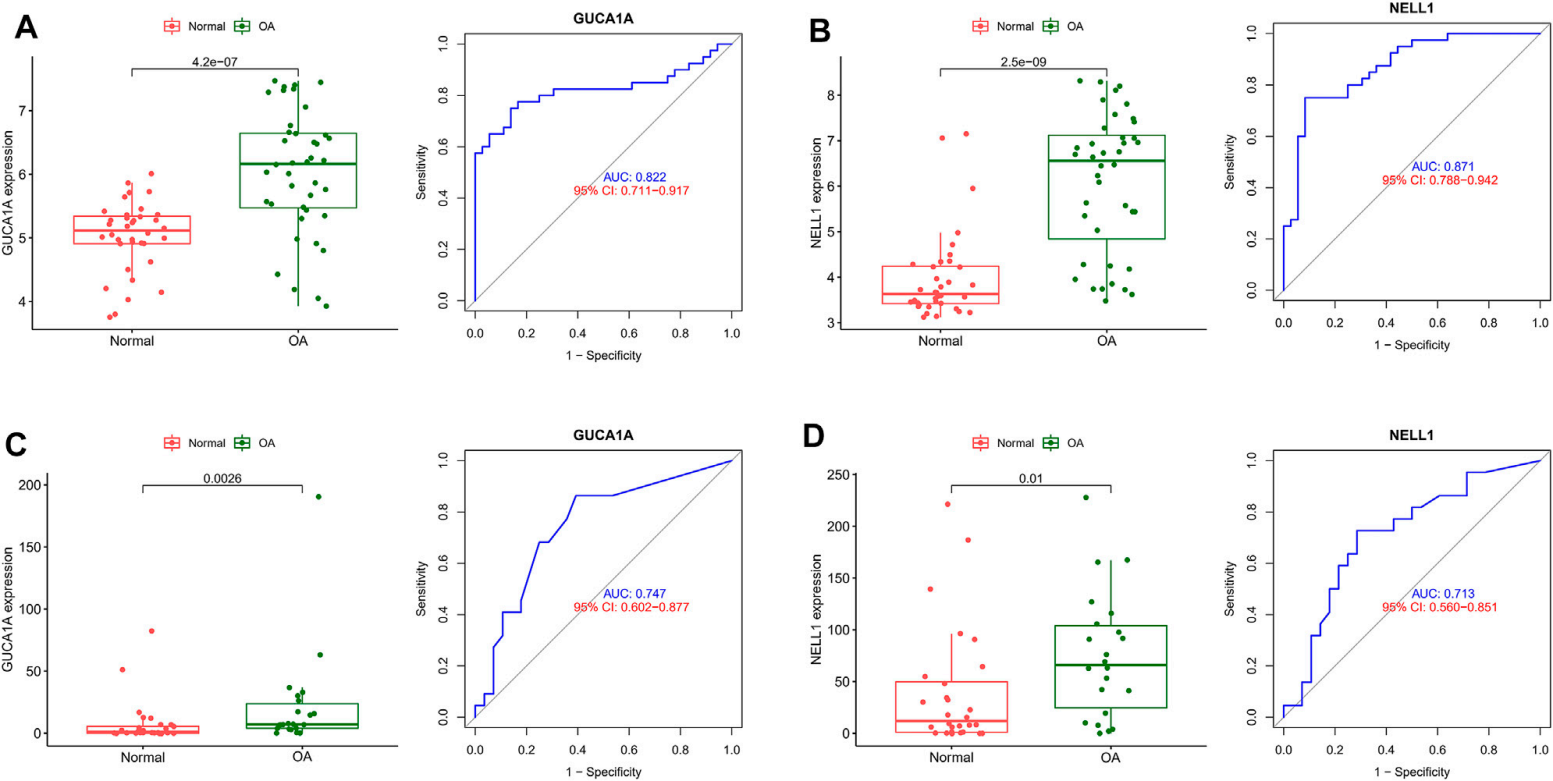
Validation of hub biomarkers. **(A)** Expression and diagnostic value of GUCA1A in OA using the test set. **(B)** Expression and diagnostic value of NELL1 in OA using the test set. **(C)** Expression and diagnostic value of GUCA1A in OA using the validation set. **(D)** Expression and diagnostic value of NELL1 in OA using the validation set.

ROC and differential expression analysis were performed on GUCA1A and NELL1, respectively. The results showed that these genes had good predictive performance in the test set: both GUCA1A (AUC = 0.822) and NELL1 (AUC = 0.871) were significantly over-expressed in the OA samples (Figures 5A,B). External validation using the GSE89408 dataset showed that the expressions of GUCA1A and NELL1 were similar to the test set, with both being up-regulated in OA tissues, and also had strong diagnostic performance (GUCA1A, AUC = 0.747; NELL1, AUC = 0.713) (Figures 5C,D). These results indicated that expressions of GUCA1A and NELL1 were highly correlated with OA progression, and that these genes may act as biomarkers to diagnose and verify effective treatment of OA.

### TF-mRNA-miRNA Network Analysis and Prediction of Potential Candidate Compounds

**FIGURE 6:**
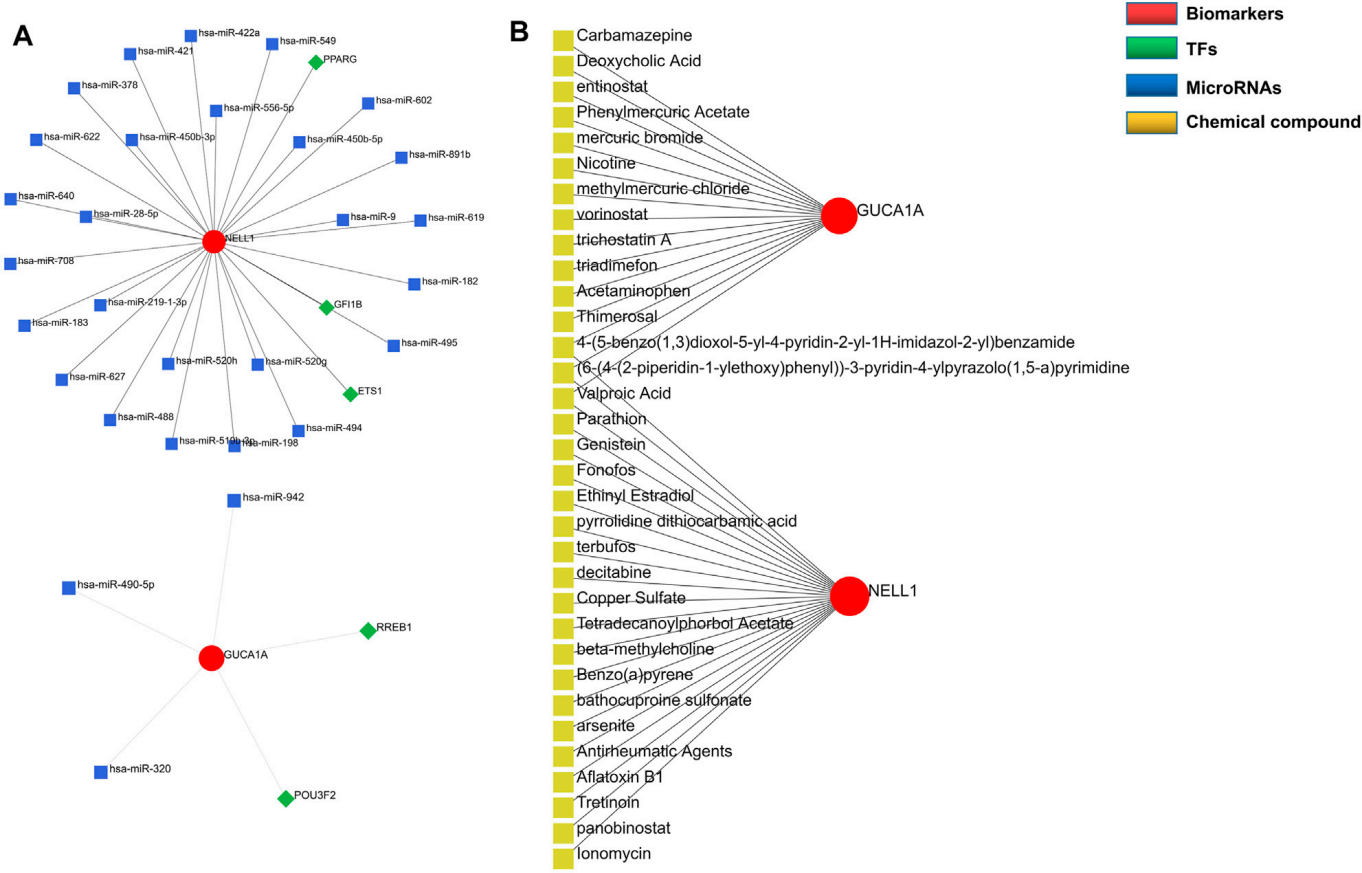
Construction of regulatory network. **(A)** TF-mRNA-miRNA network of hub biomarkers. **(B)** Potential candidate compounds targeting GUCA1A and NELL1.

Regulatory networks play a key role in understanding disease mechanisms. We used the TRRUST and mirDIP databases to predict interactions between hub biomarkers and transcription factors (TFs) as well as miRNA. A TF-mRNA-miRNA triple network was then constructed. We found 3 TFs and 26 miRNAs targeting NELL1, and identified two TF–GUCA1A pairs and three miRNA–GUCA1A pairs (Figure 6A). This network revealed hub nodes and their interactions associated with the molecular mechanisms of OA, and indicated that NELL1 and GUCA1A are related to multiple regulatory networks in OA progression. These two hub biomarkers may play a crucial role in the pathological process of OA. This enabled us to predict potential candidate compounds targeting GUCA1A and NELL1 that may alleviate OA patients’ symptoms (Figure 6B).

### Analysis of Differences in Immune Microenvironment

**FIGURE 7:**
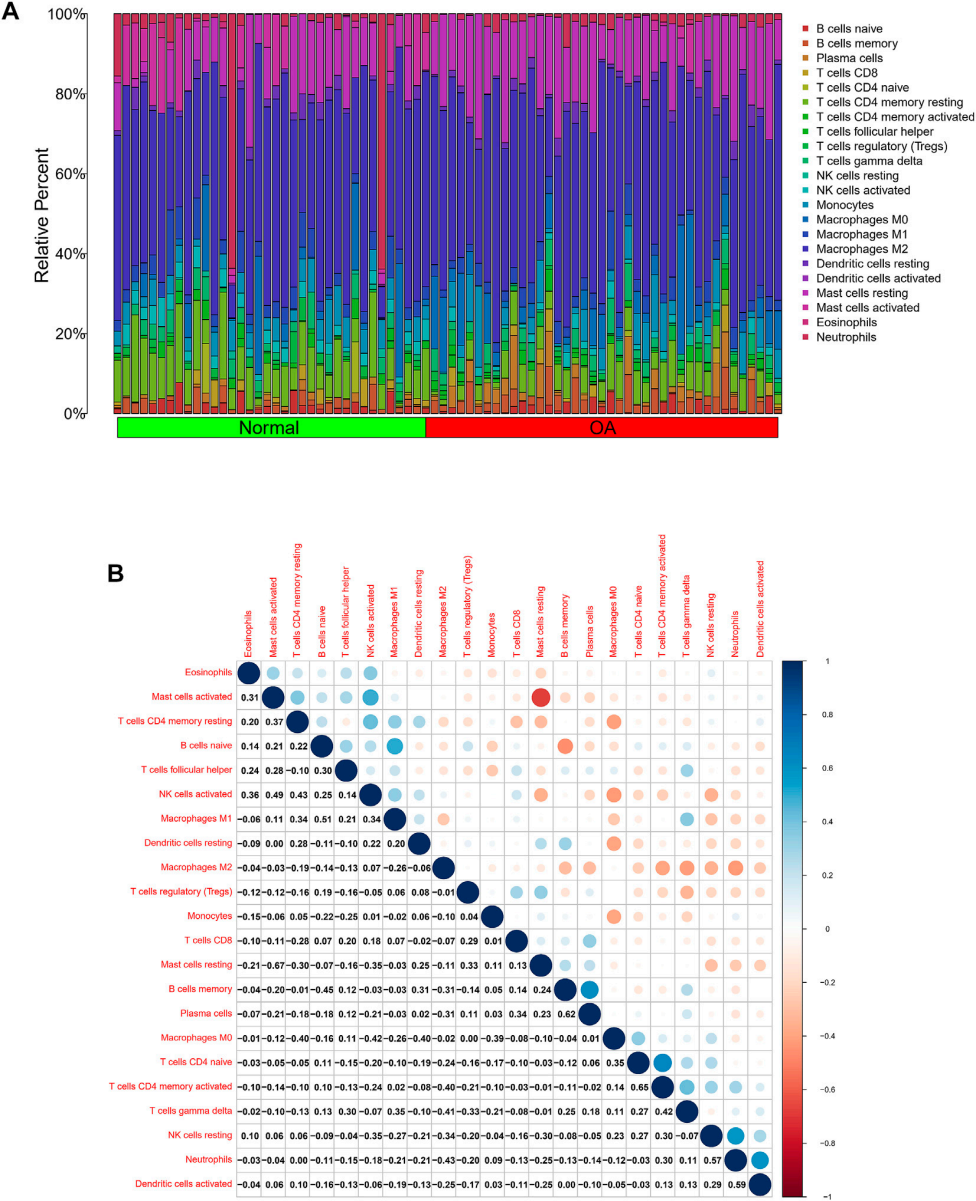
Analysis of differences in immune microenvironment. **(A)** Relative proportions of synovial tissue infiltration by 22 different immune cell subtypes. **(B)** Correlations among 22 different immune cell populations, with blue and red indicating positive and negative correlations, respectively. White indicates absence of any correlation between the indicated immune cell populations.

**FIGURE 8:**
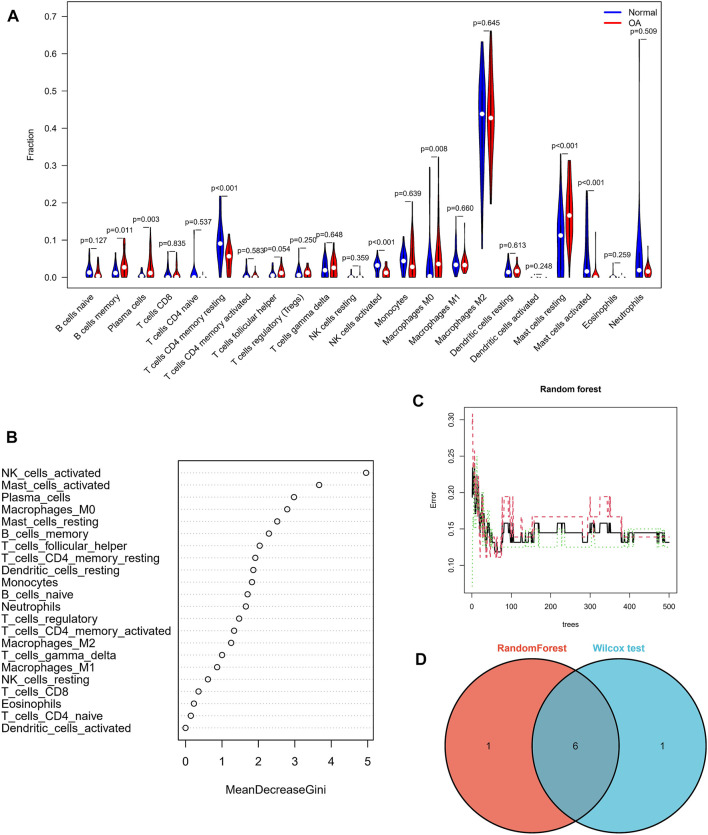
Assessment of immune cell infiltration. **(A)** Comparisons of 22 immune cell types, with blue and red indicating normal and OA tissues, respectively. **(B,C)** Random forest tree analysis of 22 immune cells. **(D)** Overlapping immune cells predicted by the immune cell and Wilcoxon test.

In view of the important role of immune-related pathways in the occurrence of OA in enrichment analysis (Figure 2), the CIBERSORT algorithm was used to analyze the content of immune cells in the different samples. The bar chart in Figure 7A illustrates the overall landscape of immune cell distribution, and the heat map in Figure 7B details the correlations of 22 immune cell types. The Wilcoxon test showed that the OA samples contained more memory B cells, plasma cells, M0 macrophages and resting mast cells. Compared with OA tissues, normal tissues had higher contents only of resting CD4 memory T cells and activated NK cells (Figure 8A). In addition, to identify hub immune cells that alter the immune microenvironment in OA synovial tissues, we performed random forest tree analysis on 22 immune cells (Figures 8B,C) and overlapped the Wilcoxon test with the immune cells identified in random forest trees. Finally, we identified six types of hub immune cells that may affect the occurrence of OA (Figure 8D): activated NK cells, activated mast cells, plasma cells, M0 macrophages, resting mast cells and memory B cells.

### Correlation Analysis of Immune Cells and Hub Biomarkers

**FIGURE 9:**
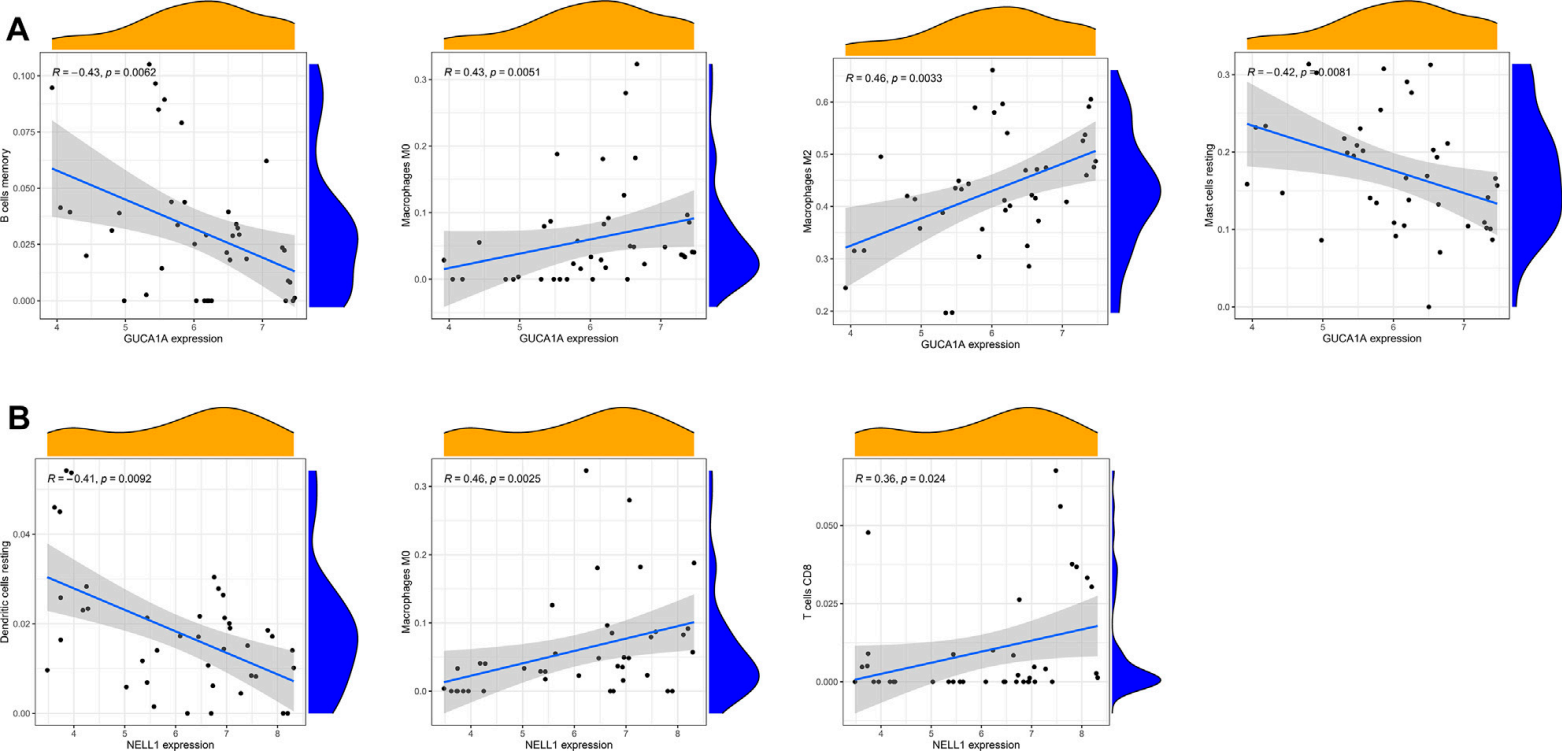
Correlation analysis of immune cells and hub biomarkers. **(A)** Correlation between GUCA1A and infiltrating immune cells. **(B)** Correlation between NELL1 and infiltrating immune cells. *p* < 0.05 considered statistically significant.

Correlation analysis between 22 kinds of immune cells and two hub biomarkers in OA tissue produced statistically significant results. GUCA1A is negatively correlated with memory B cells and resting mast cells, while M0 and M2 macrophages are positively correlated (Figure 9A). In addition, NELL1 is positively correlated with CD8 T cells and M0 macrophages, and negatively correlated with resting dendritic cells (Figure 9B).

### qRT-PCR Validation of Hub Biomarkers

**FIGURE 10:**
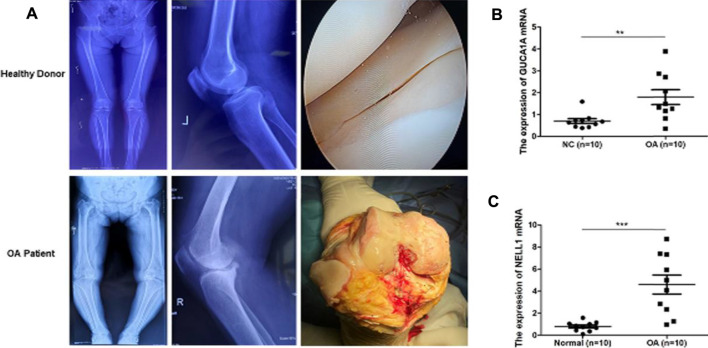
qRT-PCR validation of hub biomarkers. **(A)** X ray images and macroscopic views (arthroscopic image and intraoperative image) of knee joint from healthy donors and OA patients. **(B,C)** Validation of GUCA1A and NELL1 by qRT-PCR between the OA group (*n* = 10) and the control group (*n* = 10). Data are mean ± SEM; ∗∗*p* < 0.01, and ∗∗∗*p* < 0.001.

We performed radiological evaluations on the knee joints of different groups of patients to verify the typical imaging manifestations of OA. The results showed that compared with healthy donors, preoperative knee X-ray and gross images of OA patients showed bone spur, subchondral sclerosis and narrowing of joint space (Figure 10A). Then, qRT-PCR was used to detect the expression level of hub biomarkers in the OA synovium of the knee joint and normal control groups. Statistical analysis proved that both GUCA1A and NELL1 were significantly over-expressed in the synovium of OA samples (*p* < 0.01) (Figure 10B). All validations are consistent with the microarray hybridization, indicating that GUCA1A and NELL1 may be involved in the pathogenesis of OA.

## Discussion

OA is a chronic degenerative joint disease that causes irreversible bone erosion and cartilage destruction, and is one of the most common causes of disability (Wight et al., 2017; Smolen et al., 2018). However, because the pathophysiological mechanism of OA is unclear and effective biomarkers are lacking, diagnosis and treatment of OA is difficult. This study is the first to integrate WGCNA and machine-learning algorithms to identify new biomarkers associated with OA, and to explore the role of immune cell infiltration in OA using CIBERSORT and Wilcoxon tools.

In this study, we downloaded four gene expression profiles (GSE55235, GSE55457, GSE12021, and GSE82107) from the GEO database, and identified a total of 212 DEGs, including 102 up-regulated and 110 down-regulated genes in the OA sample compared with the normal sample. Then, we investigated the biological functions of these common DEGs and KEGG analysis revealed these genes to be enriched in the IL-17 signaling pathway and TNF signaling pathway, both of which are inflammatory. IL-17 and TNF are pro-inflammatory cytokines that are closely associated with cartilage destruction, cartilage matrix degradation and bone resorption (Kenna and Brown, 2013; Wang and He, 2018), both of which are promising therapeutic targets related to OA, which is consistent with our findings and highlights the correlation between these gene and the pathogenesis of OA. Our GO enrichment analysis of DEGs suggested that immune responses, such as regulation of leukocyte migration and myeloid leukocyte migration, were stronger in OA samples than in normal tissues. OA is a chronic inflammation disease, which closely related to immune cell infiltration of bone and cartilage (Haseeb and Haqqi, 2013), and immune cell infiltration of OA synovial tissue correlates with OA disease progression and pain (Lopes et al., 2017). These results confirm that immune-related factors may affect the progression of OA, so continued efforts to identify OA-related immune cell infiltration may be of value for treatment of this disease.

To better understand the progression of OA, hub modules and candidate biomarkers of OA were identified using WGCNA. WGCNA analysis indicated that the blue and grey modules containing 1,904 genes were most strongly correlated with clinical characteristics of OA. Using three different machine-learning algorithms (LASSO, SVM-RFE and logistic regression), GUCA1A and NELL1 were identified as hub genes, which were statistically significantly over-expressed in the OA samples (*p* < 0.05). ROC curve analysis showed that GUCA1A and NELL1 had high sensitivity and specificity in OA diagnosis in both the test set and the validation set. We also constructed a TF-mRNA-miRNA network, enabling us to predict potential candidate compounds targeting GUCA1A and NELL1 to elucidate the pathogenesis of OA at the transcriptome level. NEL-like molecule-1 (NELL1), a new secretory protein originally identified in unilateral coronal craniosynostosis in humans (Ting et al., 1999), plays an important role in osteogenic differentiation, bone regeneration, chondrogenesis and inflammation (Aghaloo et al., 2006; Lee et al., 2010; Cao et al., 2021). Recent research has reported the ability of NELL1 to induce chondrogenesis and an anti-inflammatory response in OA through up-regulation of runt-related transcription factor 1 (RUNX1), making it a potential candidate for articular cartilage repair (Li et al., 2020). In our study, compared with healthy patients, we found that the expression level of NELL1 increases in OA synovial tissues. The ROC curve for NELL1 indicated that it has good predictive performance in OA (AUC >0.87), suggesting that NELL1 plays a significant role in the progression of OA. OA is considered to be an inflammatory disease of the joint cartilage and is caused by multiple factors. Inflammatory cytokines are mainly expressed in OA, but under pathological conditions, the body still has some anti-inflammatory gene expression for self-protection and repair. When the ultimate balance between anti-inflammatory and pro-inflammatory is broken, anti-inflammatory genes can’t work as effectively (Wojdasiewicz et al., 2014). NELL1 may play this role, and the expression of NELL1 may be the body’s self-protection regulation. Li et al. found that NELL1-haploinsufficient (NELL1+/6R) mice showed elevated inflammatory markers and accelerated progression of OA. After intra-articular injection of NELL1, the IL1β-induced inflammation and cartilage degradation were rescued obviously (Li et al., 2020). The above results remind us NELL1 may be a promising target for precise treatment of OA for suppressing inflammation and arthritis-related cartilage damage. The guanylate cyclase activator 1A gene (GUCA1A), located in 6p21.1, encodes guanylyl cyclase-activating protein 1 (GCAP1), and has been identified as being involved in dominant cone dystrophy, cone-rod dystrophy and macular dystrophy (Payne et al., 1998; Wilkie et al., 2000). However, GUCA1A has not previously been reported in OA-related studies. We have identified that GUCA1A is also highly specifically expressed in the synovial membrane of OA and has a high diagnostic value (AUC >0.82) for OA. These results were validated using the GSE89408 dataset. Finally, the results of qRT-PCR showed both GUCA1A and NELL1 were significantly increased in OA samples (*p* < 0.01). All validations are consistent with the microarray hybridization, indicating that GUCA1A and NELL1 may be involved in the pathogenesis of OA, and thus we consider NELL1 and GUCA1A to be very effective biomarkers for OA diagnosis.

To explore the potential pathological relevance of immune cell infiltration in this disease, we used the CIBERSORT algorithm to conduct a comprehensive evaluation of OA immune infiltration, which provided insights into how these immune cells affect OA pathology. We found that increased infiltration of memory B cells, plasma cells, resting mast cells and M0 macrophages, and decreased infiltration of resting memory CD4 T cells and activated NK cells may be related to the occurrence and development of OA. De Lange-Brokaar et al. have found that mast cell content is significantly higher in OA samples compared with RA, and is associated with structural damage in OA patients, suggesting the role of mast cells in this disease (De Lange-Brokaar et al., 2016). Previous studies have shown that macrophages may regulate joint inflammation and OA severity through various secretory mediators, and the modulation of macrophage functional phenotypes appears to be an effective treatment option to prevent OA or enhance cartilage repair and regeneration (Wu et al., 2020; Zhang et al., 2020). Studies indicate that accumulation of memory CD4 T cells is a common phenomenon during the local inflammatory response of RA and OA joints, and is involved in the progression of OA (Ezawa et al., 1997). Increasing evidence suggests that NK cells are key to promoting immune cells in OA, and that their interaction is promoted by the CXCL10/CXCR3 axis (Benigni et al., 2017). Our analysis results combined with the above literature evidence have shown that resting mast cells, M0 macrophages, resting memory CD4 T cells and activated NK cells play important roles in OA, which should be the focus of further research. However, no research has been conducted on the role of memory B cells and plasma cells in OA, and further experimental data are required. In our study, to screen out hub immune cells that might alter the immune microenvironment in OA synovial tissues, we performed random forest tree analysis on 22 immune cells, and overlapped the Wilcoxon test with the immune cells identified in random forest trees. We have identified six types of hub immune cells that may affect the occurrence of OA: activated NK cells, activated mast cells, resting mast cells, memory B cells, plasma cells and M0 macrophages. In addition, associations between GUCA1A, NELL1 and immune cells revealed these genes to be correlated with levels of CD8 T cells, memory B cells, resting mast cells, resting dendritic cells, and M0 and M2 macrophages. We hypothesize that GUCA1A and NELL1 may be involved in the occurrence and progression of OA by mediating the above immune cells, and further studies are needed to clarify the complex interaction between genes and immune cells. The above results suggest that various infiltrating immune cells play key roles in OA pathogenesis.

Some potential limitations of this study must be considered when interpreting the results. The CIBERSORT analysis was based on limited genetic data that may deviate from heterotypic interactions of cells, disease-induced disorders or phenotypic plasticity. The exact mechanisms of NELL1 and GUCA1A in regulating the initiation and progression of OA require further investigation, and further experimental studies are needed to validate the findings of this study.

## Conclusion

In conclusion, we identify GUCA1A and NELL1 as diagnostic biomarkers of OA, and find that memory B cells, plasma cells, resting mast cells, M0 macrophages, resting CD4 memory T cells and activated NK cells may relate to the occurrence and progression of OA. These immune cells and immune-related genes may be potential immunotherapeutic targets for patients with OA.

## Data Availability

The datasets presented in this study can be found in online repositories. The names of the repository/repositories and accession number(s) can be found in the article/Supplementary Material.

## References

[B1] Aghaloo T. Cowan C. M. Chou Y.-F. Zhang X. Lee H. Miao S. (2006). Nell-1-induced Bone Regeneration in Calvarial Defects. Am. J. Pathol. 169, 903–915. 10.2353/ajpath.2006.051210 16936265 PMC1698834

[B2] Barrett T. Wilhite S. E. Ledoux P. Evangelista C. Kim I. F. Tomashevsky M. (2013). NCBI GEO: Archive for Functional Genomics Data Sets-Update. Nucleic Acids Res. 41, D991–D995. 10.1093/nar/gks1193 23193258 PMC3531084

[B3] Benigni G. Dimitrova P. Antonangeli F. Sanseviero E. Milanova V. Blom A. (2017). CXCR3/CXCL10 axis Regulates Neutrophil-NK Cell Cross-Talk Determining the Severity of Experimental Osteoarthritis. J.I. 198, 2115–2124. 10.4049/jimmunol.1601359 28108560

[B4] Cao R. Wang Q. Wu J. Liu M. Han Q. Wang X. (2021). Nell-1 Attenuates Lipopolysaccharide-Induced Inflammation in Human Dental Pulp Cells. J. Mol. Histol. 52, 671–680. 10.1007/s10735-021-09976-y 33905072

[B5] Carr H. L. Turner J. D. Major T. Scheel-Toellner D. Filer A. (2020). New Developments in Transcriptomic Analysis of Synovial Tissue. Front. Med. 7, 21. 10.3389/fmed.2020.00021 PMC7005068 32083090

[B6] Chen B. Khodadoust M. S. Liu C. L. Newman A. M. Alizadeh A. A. (2018). Profiling Tumor Infiltrating Immune Cells with CIBERSORT. Methods Mol. Biol. 1711, 243–259. 10.1007/978-1-4939-7493-1_12 29344893 PMC5895181

[B7] Cheng Q. Chen X. Wu H. Du Y. (2021). Three Hematologic/immune System-specific Expressed Genes Are Considered as the Potential Biomarkers for the Diagnosis of Early Rheumatoid Arthritis through Bioinformatics Analysis. J. Transl Med. 19, 18. 10.1186/s12967-020-02689-y 33407587 PMC7789535

[B8] Davis A. P. Grondin C. J. Johnson R. J. Sciaky D. McMorran R. Wiegers J. (2019). The Comparative Toxicogenomics Database: Update 2019. Nucleic Acids Res. 47, D948–D954. 10.1093/nar/gky868 30247620 PMC6323936

[B9] De Lange-Brokaar B. J. E. Kloppenburg M. Andersen S. N. Dorjée A. L. Yusuf E. Herb-van Toorn L. (2016). Characterization of Synovial Mast Cells in Knee Osteoarthritis: Association with Clinical Parameters. Osteoarthritis and Cartilage 24, 664–671. 10.1016/j.joca.2015.11.011 26671522

[B10] Demircioğlu D. Cukuroglu E. Kindermans M. Nandi T. Calabrese C. Fonseca N. A. (2019). A Pan-Cancer Transcriptome Analysis Reveals Pervasive Regulation through Alternative Promoters. Cell 178, 1465–1477. 10.1016/j.cell.2019.08.018 31491388

[B11] Engebretsen S. Bohlin J. (2019). Statistical Predictions with Glmnet. Clin. Epigenet 11, 123. 10.1186/s13148-019-0730-1 PMC6708235 31443682

[B12] Ezawa K. Yamamura M. Matsui H. Ota Z. Makino H. (1997). Comparative Analysis of CD45RA- and CD45RO-Positive CD4+T Cells in Peripheral Blood, Synovial Fluid, and Synovial Tissue in Patients with Rheumatoid Arthritis and Osteoarthritis. Acta Med. Okayama 51, 25–31. 10.18926/amo/30810 9057932

[B13] Han H. Cho J.-W. Lee S. Yun A. Kim H. Bae D. (2018). TRRUST V2: an Expanded Reference Database of Human and Mouse Transcriptional Regulatory Interactions. Nucleic Acids Res. 46, D380–D386. 10.1093/nar/gkx1013 29087512 PMC5753191

[B14] Hänzelmann S. Castelo R. Guinney J. (2013). GSVA: Gene Set Variation Analysis for Microarray and RNA-Seq Data. BMC Bioinformatics 14, 7. 10.1186/1471-2105-14-7 23323831 PMC3618321

[B15] Haseeb A. Haqqi T. M. (2013). Immunopathogenesis of Osteoarthritis. Clin. Immunol. 146, 185–196. 10.1016/j.clim.2012.12.011 23360836 PMC4015466

[B16] Hootman J. M. Helmick C. G. (2006). Projections of US Prevalence of Arthritis and Associated Activity Limitations. Arthritis Rheum. 54, 226–229. 10.1002/art.21562 16385518

[B17] Kenna T. J. Brown M. A. (2013). The Role of IL-17-secreting Mast Cells in Inflammatory Joint Disease. Nat. Rev. Rheumatol. 9, 375–379. 10.1038/nrrheum.2012.205 23229447

[B18] Langfelder P. Horvath S. (2008). WGCNA: an R Package for Weighted Correlation Network Analysis. BMC Bioinformatics 9, 559. 10.1186/1471-2105-9-559 19114008 PMC2631488

[B19] Lee M. Siu R. K. Ting K. Wu B. M. (2010). Effect of Nell-1 Delivery on Chondrocyte Proliferation and Cartilaginous Extracellular Matrix Deposition. Tissue Eng. A 16, 1791–1800. 10.1089/ten.tea.2009.0384 20028218

[B20] Leek J. T. Johnson W. E. Parker H. S. Jaffe A. E. Storey J. D. (2012). The Sva Package for Removing Batch Effects and Other Unwanted Variation in High-Throughput Experiments. Bioinformatics 28, 882–883. 10.1093/bioinformatics/bts034 22257669 PMC3307112

[B21] Li C. Zheng Z. Ha P. Jiang W. Berthiaume E. A. Lee S. (2020). Neural EGFL like 1 as a Potential Pro-chondrogenic, Anti-inflammatory Dual-Functional Disease-Modifying Osteoarthritis Drug. Biomaterials 226, 119541. 10.1016/j.biomaterials.2019.119541 31634652 PMC6938239

[B22] Li M. H. Xiao R. Li J. B. Zhu Q. (2017). Regenerative Approaches for Cartilage Repair in the Treatment of Osteoarthritis. Osteoarthritis and Cartilage 25, 1577–1587. 10.1016/j.joca.2017.07.004 28705606

[B23] Liberzon A. Birger C. Thorvaldsdóttir H. Ghandi M. Mesirov J. P. Tamayo P. (2015). The Molecular Signatures Database Hallmark Gene Set Collection. Cel Syst. 1, 417–425. 10.1016/j.cels.2015.12.004 PMC4707969 26771021

[B24] Lin X. Yang F. Zhou L. Yin P. Kong H. Xing W. (2012). A Support Vector Machine-Recursive Feature Elimination Feature Selection Method Based on Artificial Contrast Variables and Mutual Information. J. Chromatogr. B 910, 149–155. 10.1016/j.jchromb.2012.05.020 22682888

[B25] Lopes E. B. P. Filiberti A. Husain S. A. Humphrey M. B. (2017). Immune Contributions to Osteoarthritis. Curr. Osteoporos. Rep. 15, 593–600. 10.1007/s11914-017-0411-y 29098574

[B26] Mathiessen A. Conaghan P. G. (2017). Synovitis in Osteoarthritis: Current Understanding with Therapeutic Implications. Arthritis Res. Ther. 19, 18. 10.1186/s13075-017-1229-9 28148295 PMC5289060

[B27] Parkinson L. Waters D. L. Franck L. (2017). Systematic Review of the Impact of Osteoarthritis on Health Outcomes for Comorbid Disease in Older People. Osteoarthritis and Cartilage 25, 1751–1770. 10.1016/j.joca.2017.07.008 28710026

[B28] Payne A. Downes S. M. Bessant D. A. Taylor R. Holder G. E. Warren M. J. (1998). A Mutation in Guanylate Cyclase Activator 1A (GUCA1A) in an Autosomal Dominant Cone Dystrophy Pedigree Mapping to a New Locus on Chromosome 6p21.1. Hum. Mol. Genet. 7, 273–277. 10.1093/hmg/7.2.273 9425234

[B29] Ritchie M. E. Phipson B. Wu D. Hu Y. Law C. W. Shi W. (2015). *Limma* powers Differential Expression Analyses for RNA-Sequencing and Microarray Studies. Nucleic Acids Res. 43, e47. 10.1093/nar/gkv007 25605792 PMC4402510

[B30] Robin X. Turck N. Hainard A. Tiberti N. Lisacek F. Sanchez J.-C. (2011). pROC: an Open-Source Package for R and S+ to Analyze and Compare ROC Curves. BMC Bioinformatics 12, 7. 10.1186/1471-2105-12-77 21414208 PMC3068975

[B31] Seed S. M. Dunican K. C. Lynch A. M. (2011). Treatment Options for Osteoarthritis: Considerations for Older Adults. Hosp. Pract. 39, 62–73. 10.3810/hp.2011.02.375 21441760

[B32] Smolen J. S. Aletaha D. Barton A. Burmester G. R. Emery P. Firestein G. S. (2018). Rheumatoid Arthritis. Nat. Rev. Dis. Primers 4, 18001. 10.1038/nrdp.2018.1 29417936

[B33] Tian R. Zhang S. Sun D. Bei C. Li D. Zheng C. (2020). M6A Demethylase FTO Plays a Tumor Suppressor Role in Thyroid Cancer. DNA Cel Biol. 39, 2184–2193. 10.1089/dna.2020.5956 33054406

[B34] Ting K. Vastardis H. Mulliken J. B. Soo C. Tieu A. Do H. (1999). Human Nell-1 Expressed in Unilateral Coronal Synostosis. J. Bone Miner Res. 14, 80–89. 10.1359/jbmr.1999.14.1.80 9893069

[B35] Tokar T. Pastrello C. Rossos A. E. M. Abovsky M. Hauschild A.-C. Tsay M. (2018). mirDIP 4.1-integrative Database of Human microRNA Target Predictions. Nucleic Acids Res. 46, D360–D370. 10.1093/nar/gkx1144 29194489 PMC5753284

[B36] Walter W. Sánchez-Cabo F. Ricote M. (2015). GOplot: an R Package for Visually Combining Expression Data with Functional Analysis: Fig. 1. Bioinformatics 31, 2912–2914. 10.1093/bioinformatics/btv300 25964631

[B37] Wang T. He C. (2018). Pro-inflammatory Cytokines: The Link between Obesity and Osteoarthritis. Cytokine Growth Factor. Rev. 44, 38–50. 10.1016/j.cytogfr.2018.10.002 30340925

[B38] Wight L. Owen D. Goldbloom D. Knupp M. (2017). Pure Ankle Dislocation: a Systematic Review of the Literature and Estimation of Incidence. Injury 48, 2027–2034. 10.1016/j.injury.2017.08.011 28826653

[B39] Wilkie S. E. Newbold R. J. Deery E. Walker C. E. Stinton I. Ramamurthy V. (2000). Functional Characterization of Missense Mutations at Codon 838 in Retinal Guanylate Cyclase Correlates with Disease Severity in Patients with Autosomal Dominant Cone-Rod Dystrophy. Hum. Mol. Genet. 9, 3065–3073. 10.1093/hmg/9.20.3065 11115851

[B40] Wojdasiewicz P. Poniatowski Ł. A. Szukiewicz D. (2014). The Role of Inflammatory and Anti-inflammatory Cytokines in the Pathogenesis of Osteoarthritis. Mediators Inflamm. 2014, 1–19. 10.1155/2014/561459 PMC4021678 24876674

[B41] Wu C.-L. Harasymowicz N. S. Klimak M. A. Collins K. H. Guilak F. (2020). The Role of Macrophages in Osteoarthritis and Cartilage Repair. Osteoarthritis and Cartilage 28, 544–554. 10.1016/j.joca.2019.12.007 31926267 PMC7214213

[B42] Yoon S. Kim S. (2009). AdaBoost-based Multiple SVM-RFE for Classification of Mammograms in DDSM. BMC Med. Inform. Decis. Mak 9, S1. 10.1186/1472-6947-9-S1-S1 19891795 PMC2773916

[B43] Yu G. Wang L.-G. Han Y. He Q.-Y. (2012). clusterProfiler: an R Package for Comparing Biological Themes Among Gene Clusters. OMICS: A J. Integr. Biol. 16, 284–287. 10.1089/omi.2011.0118 PMC3339379 22455463

[B44] Zhang H. Cai D. Bai X. (2020). Macrophages Regulate the Progression of Osteoarthritis. Osteoarthritis and Cartilage 28, 555–561. 10.1016/j.joca.2020.01.007 31982565

[B45] Zhao X. Zhang L. Wang J. Zhang M. Song Z. Ni B. (2021). Identification of Key Biomarkers and Immune Infiltration in Systemic Lupus Erythematosus by Integrated Bioinformatics Analysis. J. Transl Med. 19, 35. 10.1186/s12967-020-02698-x 33468161 PMC7814551

[B46] Zhou G. Soufan O. Ewald J. Hancock R. E. W. Basu N. Xia J. (2019). NetworkAnalyst 3.0: a Visual Analytics Platform for Comprehensive Gene Expression Profiling and Meta-Analysis. Nucleic Acids Res. 47, W234–W241. 10.1093/nar/gkz240 30931480 PMC6602507

