# Supplementary figures and images for: Evaluation of Biomarkers and Immune Microenvironment of Osteoarthritis: Evidence From Omics Data and Machine Learning
Source: Front Genet. 2022 May 16;13:905027. doi: 10.3389/fgene.2022.905027 (PMC9149375; doi:10.3389/fgene.2022.905027)

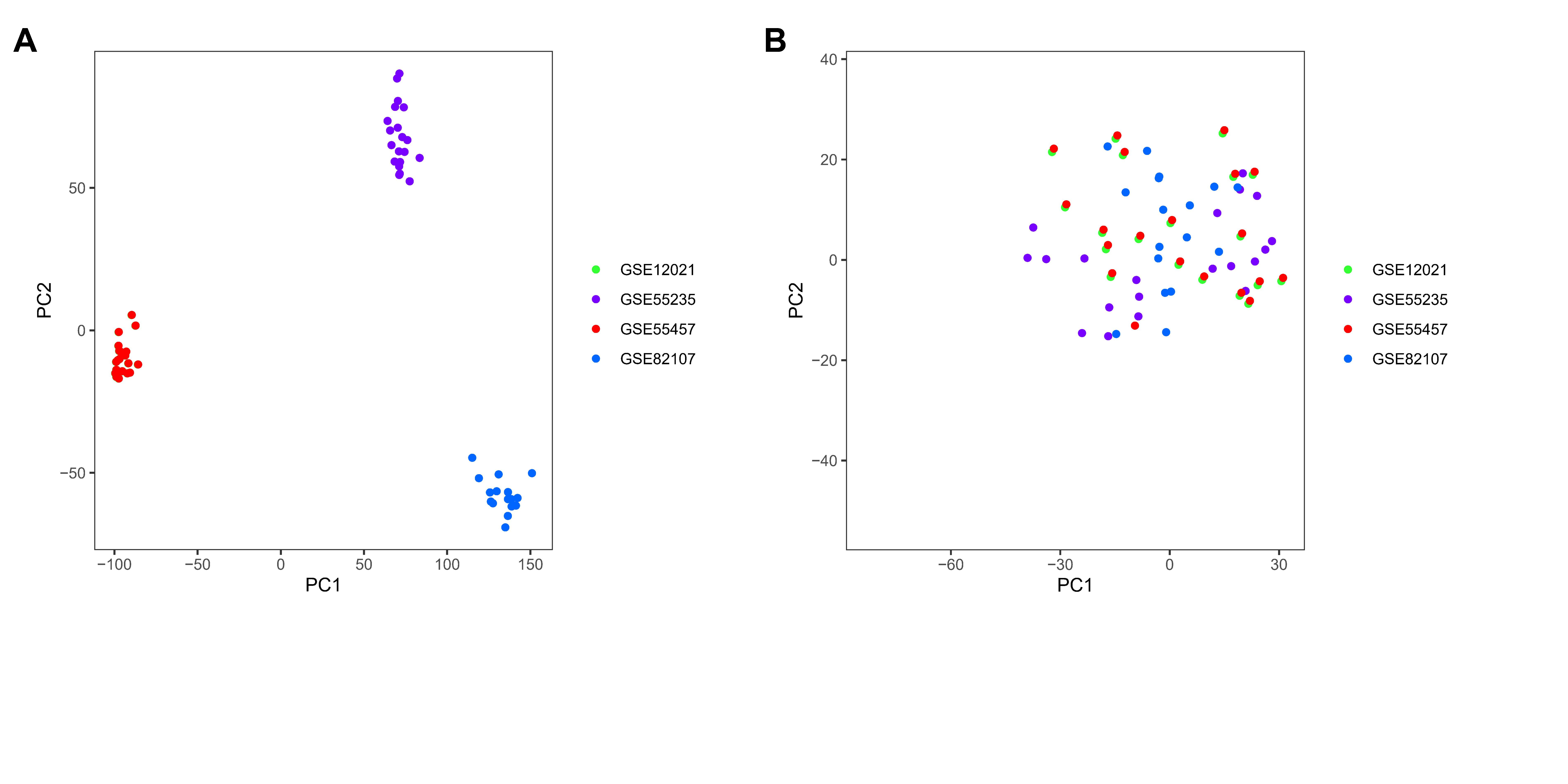

Supplement: Supplementary file 1 [file Image1.tiff]
